# Biocompatibility and Physiological Thiolytic Degradability of Radically Made Thioester-Functional Copolymers: Opportunities for Drug Release

**DOI:** 10.1021/acs.biomac.2c00039

**Published:** 2022-04-26

**Authors:** Nathaniel M. Bingham, Qamar un Nisa, Priyanka Gupta, Neil P. Young, Eirini Velliou, Peter J. Roth

**Affiliations:** † Department of Chemistry, School of Chemistry and Chemical Engineering, University of Surrey, Guildford, Surrey GU2 7XH, United Kingdom; ‡ Department of Chemical and Process Engineering, School of Chemistry and Chemical Engineering, University of Surrey, Guildford, Surrey GU2 7XH, United Kingdom; § Holder Building, Department of Materials, University of Oxford, Parks Road, Oxford OX1 3PH, United Kingdom; ∥ Centre for 3D Models of Health and Disease, UCL-Division of Surgery and Interventional Science, Charles Bell House, 43−45 Foley Street, Fitzrovia, London W1W 7TY, United Kingdom

## Abstract

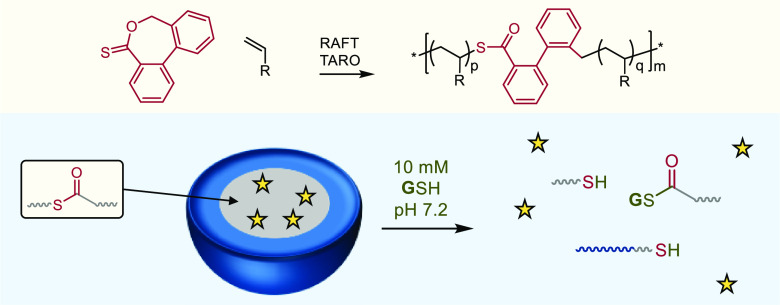

Being nondegradable, vinyl polymers have limited biomedical applicability. Unfortunately, backbone esters incorporated through conventional radical ring-opening methods do not undergo appreciable abiotic hydrolysis under physiologically relevant conditions. Here, PEG acrylate and di(ethylene glycol) acrylamide-based copolymers containing backbone thioesters were prepared through the radical ring-opening copolymerization of the thionolactone dibenzo[c,e]oxepin-5(7*H*)-thione. The thioesters degraded fully in the presence of 10 mM cysteine at pH 7.4, with the mechanism presumed to involve an irreversible S–N switch. Degradations with *N*-acetylcysteine and glutathione were reversible through the thiol–thioester exchange polycondensation of R–SC(=O)–polymer–SH fragments with full degradation relying on an increased thiolate/thioester ratio. Treatment with 10 mM glutathione at pH 7.2 (mimicking intracellular conditions) triggered an insoluble–soluble switch of a temperature-responsive copolymer at 37 °C and the release of encapsulated Nile Red (as a drug model) from core-degradable diblock copolymer micelles. Copolymers and their cysteinolytic degradation products were found to be noncytotoxic, making thioester backbone-functional polymers promising for drug delivery applications.

## Introduction

Reversible deactivation (“controlled”) radical polymerization (RDRP) methods enable the preparation of functional vinyl (co)polymers and well-defined nanostructured materials with unparalleled potential in biomedical applications.^1−4^ However, with an all-carbon backbone, vinyl copolymers are not degradable, preventing their application as degradable biomaterials, a field dominated by materials made wholly or partially^5−8^ through step-growth polycondensation reactions or ionic ring-opening polymerization^9,10^ (which can provide a degradable moiety in every repeat unit but lack the diversity and functionality of vinyl comonomers and/or architectural control of radical methods).

The incorporation of (partial) degradability into vinyl copolymers is thus considered a remaining frontier in the biomedical arena with many applications (tissue engineering, drug release, drug delivery with renal clearance of polymer fragments) bound to benefit.^11^ To date, the most common way of conveying backbone degradability to vinyl copolymers is the introduction of backbone esters through the radical ring-opening polymerization (RROP)^12^ of cyclic ketene acetals^13,14^ or allyl sulfide lactones.^15−18^ While the enzymatic degradation of these ester groups has been demonstrated on several systems, for example, using fungal^19^ or bacterial^20,21^ lipases, proteinase,^22^ or pig liver esterase,^17^ these conditions are not representative of other biological environments. Hydrolysis is commonly achieved using NaOH or KOH (up to 10 wt %) in water or methanol,^23−25^ with abiotic hydrolysis under biologically relevant conditions observed after months.^26^ Irrespective of the degradation method, it is usually not possible to selectively cleave backbone esters in the presence of side group esters, for example, of (meth)acrylic or vinyl ester comonomer units. During the writing of this article, Nicolas and co-workers^27^ presented an acrylamide–cyclic ketene acetal copolymer^28^ with backbone esters capable of hydrolytic degradation over the course of several days.

More elaborate approaches toward vinyl copolymer degradability have therefore involved the incorporation of weaker linkages with a major emphasis on the disulfide functionality based on the potential of reductive cleavage through intracellular glutathione.^29,30^ The radical ring-opening copolymerization of a disulfide-functional allyl sulfide lactone with methacrylate comonomers,^17^ for example, provided ester and disulfide backbone functionality. Other approaches have involved the use of a difunctional disulfide initiator (providing one cleavable unit in the center of a (co)polymer)^31^ and the step-growth polycondensations of α,ω-functional prepolymers through S–S coupling^7^ or thiol oxidation.^32^

Recently, thiocarbonyl addition–ring-opening (TARO)^33^ radical polymerization of thionolactones was shown to introduce *thio*ester backbone units into acrylate-,^33,34^ acrylamide-,^33,35^ maleimide-,^36^ styrene,^37^ and vinyl ester-^38,39^ based polymers. The method is compatible with reversible addition–fragmentation chain transfer (RAFT) radical polymerization (a major RDRP method) and thus enables the preparation of degradable copolymers with controlled architectures, including block copolymers.^34,39^ Thioesters are important reactive intermediates in many biological processes including protein, carbohydrate, and lipid metabolism.^40^ Thioester-functional polymers^41^ including those made through nonradical processes,^42,43^ are receiving increasing interest including as smart materials,^35^ for reversible PEGylation,^44^ or recycling,^45^ but their degradability, including under physiologically relevant conditions or potential for drug delivery, has not been systematically assessed.

Here, we demonstrate that the thioester linkages within PEG-based copolymers are weak enough to be degraded by the biologically relevant thiols cysteine, *N*-acetyl cysteine, and glutathione (including under physiological conditions). Differences in the degradation mechanisms and a “reversible degradation” process leading to the formation of larger species than the intact polymer are discussed. The glutathione degradation is shown to trigger a solubility switch of a thermoresponsive copolymer and the (partial) release of an encapsulated dye from a diblock copolymer micelle. Finally, thioester-functional copolymers and their degradation products are shown to be noncytotoxic up to polymer concentrations of 1 g/L.

## Experimental Section

Details on instrumentation, materials, and the synthesis of DEGAm are given in the Supporting Information. The synthesis of the thionolactone dibenzo[c,e]oxepin-5(7*H*)-thione (DOT) and the RAFT agent *S*-benzyl-*S*′-propyl trithiocarbonate were previous described.^36^

### General Polymerization Procedure

DOT and comonomer (in varying molar ratios as described below), *S*-benzyl-*S*′-propyl trithiocarbonate (1 equiv), AIBN (0.25 equiv), and DMSO (total monomer conc. = 3.3 M) were added into a ground-glass joint tube. The reaction was sealed with a rubber septum, stirred and degassed with nitrogen for 30 min through a needle with a shorter needle fitted for gas release. The tube was placed in a preheated oil bath set at 80 °C and left for a predetermined amount of time. After cooling and exposing to air, the monomer conversion was determined by ^1^H NMR spectroscopy of the crude mixture diluted with CDCl_3_. Copolymers were purified by dialysis against methanol, then water in regenerated cellulose membranes (3500 g mol^–1^ molecular weight cutoff), followed by freeze-drying.

### Degradation Procedures

Degradant solutions were prepared by dissolving thiol (cysteine, *N*-acetylcysteine, glutathione; 10 or 100 mM) and tris(carboxyethyl phosphine) (TCEP, as reducing agent to prevent disulfide formation, 1 mM or 10 mM, respectively) in phosphate buffered saline, followed by dropwise addition of 300 mM NaOH and additional buffer to adjust the pH to 7.2 (for glutathione solutions) or 7.4 (for cysteine and *N*-acetylcysteine solutions) and adjust the final concentrations as indicated above. P(PEGA_218_-DOT_22_) (5.0 mg, containing 1.0 μmol of thioesters) was dissolved in degradant solutions (2 mL of 100 mM (or 10 mM) solution for a final ratio of 200 thiols (or 20 thiols) per thioester or 39 mL of 10 mM glutathione solution for a final ratio of 390 thiols per thioester). The polymer dissolved quickly (within 1–2 min) and the solutions were stirred at 37 °C under nitrogen for a predetermined amount of time. The mixture was extracted with dichloromethane (2× 4 mL for 2 mL-sized experiments; 2× 30 mL for 39 mL-sized experiment). The combined organic extracts were dried (MgSO_4_), filtered, and evaporated to dryness. The residual material (typically 3–4 mg) was dissolved in THF and analyzed by SEC.

For comparison, copolymers were degraded in 7 M NH_3_ in methanol, previously judged to lead to complete degradation.^35^ Copolymer (4 mg) was dissolved in 7 M NH_3_ in methanol, and the solution stirred overnight in a closed container at RT. A stream of air was blown into the solution until all volatiles had evaporated and no smell of ammonia remained. The residual material was dissolved in THF and analyzed by SEC.

Degradation with potassium persulfate was done by adding aqueous oxone solution (100 mM, 111 μL) to polymer solution (1 mL) to achieve a final oxone concentration of 10 mM.

### 2D Cell Culture

The human pancreatic adenocarcinoma cell line PANC-1 (Sigma-Aldrich, U.K.) was expanded in Dulbecco’s modified Eagle’s medium (DMEM) with high glucose (Lonza, U.K.) supplemented with 10% fetal bovine serum (Fisher Scientific, U.K.), 1% penicillin/streptomycin (Fisher Scientific, U.K.) and 2 mM l-glutamine (Sigma-Aldrich, U.K.) in a humidified incubator at 37 °C with 5% CO_2_ and above-90% relative humidity. Cells were passaged regularly on reaching 90% confluency until the required cell density was obtained. Assessment of the polymer cytocompatibility and toxicity was then carried out over a period of 72 h. Briefly, 1000 PANC-1 cells were seeded per well in 48-well plates with polymer concentrations of 0.001, 0.01, 0.1, 1, or 10 g/L. The cells were incubated in a humidified incubator at 37 °C with 5% CO_2_ for 72 h. Experiments were performed in triplicate.

### Alamar Blue Assay

An Alamar Blue cell viability assay was carried out after 48 and 72 h to assess cell viability and growth as a direct measure of the cytocompatibility of the polymers. A 10% Alamar Blue solution (Thermo Fisher, U.K.) in complete cell culture medium was added to the culture and incubated for 2 h. At the end of the incubation period, the change in Alamar Blue fluorescence was measured using a BioTek, Plate reader (BioTek, U.K.) at 530 nm excitation and 590 nm emission. Further measurement of change in fluorescence was carried out after 72 h post seeding to assess cytocompatibility of the polymers over time.

## Results and Discussion

### Synthesis

**Scheme 1:**
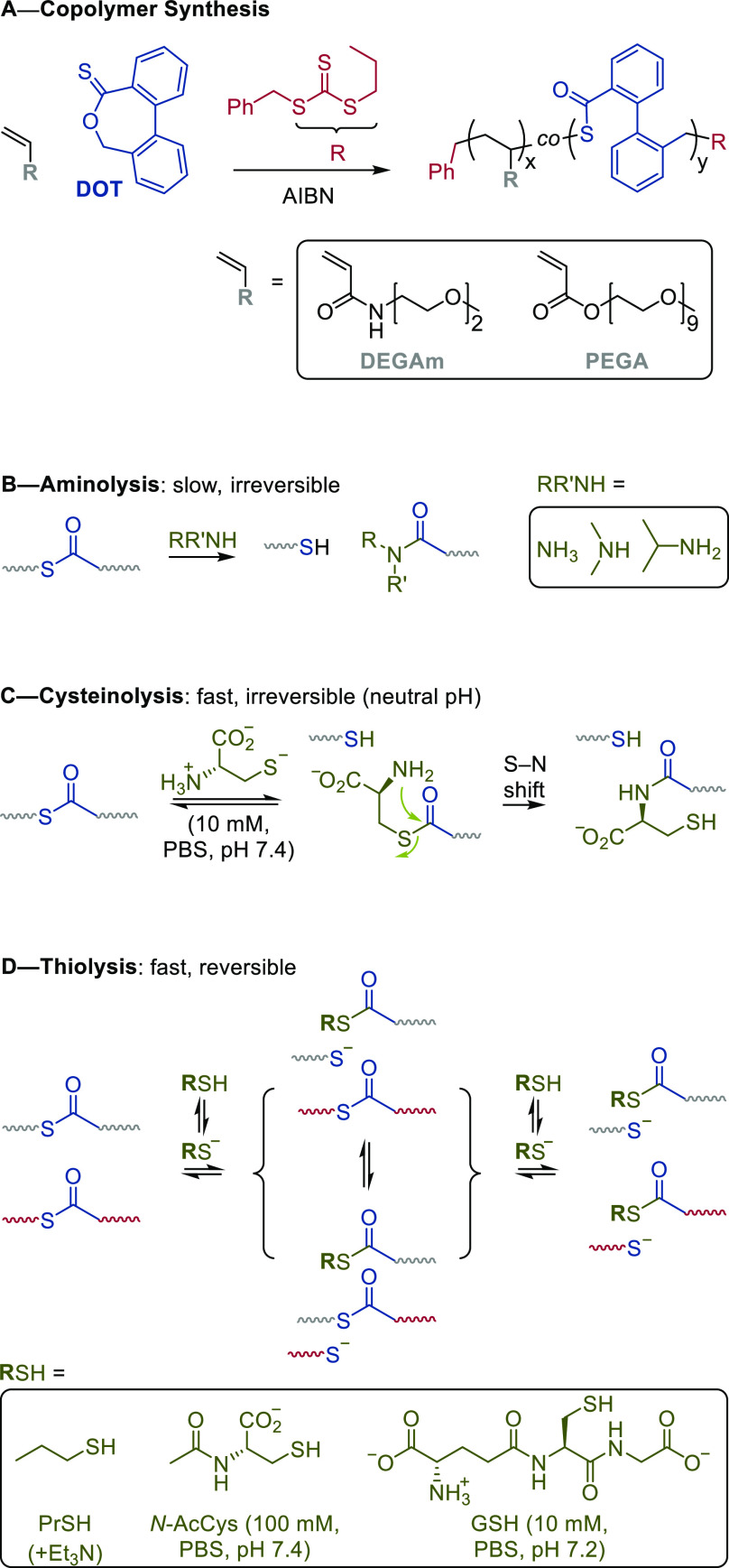
Synthesis of Degradable Thioester-Functional Acrylate and Acrylamide Copolymers Via RAFT thiocarbonyl addition–ring-opening (TARO) radical copolymerization (A) and reagents and mechanisms for the degradation of thioester-functional polymers through aminolysis (B), cysteinolysis (C), and thiolysis (D).

Thioester backbone-functional copolymers were prepared through reversible addition–fragmentation chain transfer (RAFT) thiocarbonyl addition–ring-opening (TARO) radical copolymerization of the thionolactone dibenzo[c,e]oxepine-5(7*H*)-thione (DOT) with one of two vinyl comonomers, see Scheme 1A. Poly(ethylene glycol) methyl ether acrylate (PEGA) was chosen as a well-documented monomer of biocompatible polymers.^46^ As an acrylamide representative, di(ethylene glycol) methyl ether acrylamide (DEGAm) was prepared. While its polymer, pDEGAm, prepared through postpolymerization modification of an activated ester precursor, had been shown to be noncytotoxic,^47^ the polymerization of the monomer DEGAm, as shown here, to the best of our knowledge has not been reported. Two homo- and six copolymers were investigated; see Table 1. The molar DOT content was kept between 2 and 15.6 mol % to ensure water-solubility at room temperature.^35^

**Table 1:** Overview of Homo-and Thioester-Functional Copolymers Used in This Study^a^

entry	composition^b^	DOT content^b^ (mol %)	comonomer feed (equiv)	*M*_n_^NMR^^b^ (kg/mol)	*M*_n_^SEC^ (kg/mol)	*Đ*^SEC^	water solubility
1	p(PEGA_247_)	0	250 + 0	119	11.5	1.36	soluble
2	p(PEGA_243_-DOT_5_)	2.0	245 + 5	118	14.1	1.32	soluble
3	p(PEGA_235_-DOT_11_)	4.5	237.5 + 12.5	117	16.0	1.28	soluble
4	p(PEGA_218_-DOT_22_)	9.2	225 + 25	110	20.7	1.24	soluble
5	p(DEGAm_206_-DOT_5_)	2.4	245 + 5	36.8	n.d.^c^	n.d.	soluble
6	p(DEGAm_238_)	0	250 + 0	41.0	n.d.^c^	n.d.	soluble
7	p(DEGAm_226_-DOT_13_)	5.4	237 + 13	41.9	n.d.^c^	n.d.	soluble
8	p(DEGAm_27_-DOT_5_)^d^	15.6	95 + 5	5.64	2.45	1.22	LCST 36 °C

a Polymerization time 16 h at 80 °C.

b Molar comonomer content and molar mass calculated from monomer conversion determined by ^1^H NMR spectroscopy.

c Sample provided insufficient RI contrast to detect in SEC measurement.

d Polymerization time = 0.5 h to achieve lower critical solution temperature.^35^

### Degradability

Next, the degradation behavior of DOT–PEGA copolymers in water, phosphate buffered saline (PBS, pH = 7.4) and in the presence of various degradants was assessed using size exclusion chromatography (SEC). Because of a higher reactivity of the thionolactone compared to acrylate and acrylamide comonomers, their RAFT copolymerization (when taken to near-completion) produces gradient copolymers that have a higher concentration of degradable linkages at the beginning of chains. If all thioesters (or at least the one closest to the undegradable homovinyl “tail”) are cleaved, typically only the large undegradable section is visible through refractive index detection in SEC analysis while the shorter fragments are not (due to lower concentration, a lower refractive index increment,^48^ and potential loss during workup). On the other hand, incomplete degradation will produce (at least some) tail sections still carrying thioester-connected portions of the original chain, apparent through a shift (or shoulder) of the SEC-measured curve toward higher apparent molar masses.

Previously, our group showed that backbone thioesters did not degrade during dialysis against methanol and water. Conversely, in the presence of amines, thioesters slowly underwent aminolysis (Scheme 1B), requiring a large amine/thioester excess to achieve (partial) degradation after 1 day with complete degradation found for 7 M NH_3_ in MeOH.^35^ Here, the stability of DOT-derived backbone thioesters in water and PBS was confirmed. After dissolving p(PEGA_243_-DOT_5_) (Table 1, entry 2) in water or PBS for 1 week at RT, SEC analysis showed no significant change (Figure S1A). Similarly, after being dissolved in water for 56 days, the SEC trace of p(PEGA_235_-DOT_11_) (Table 1 entry 3) showed only a very minor increase of the amount of smaller species with no shift of the larger molar mass flank of the elution curve (Figure S1B). The hydrolysis half-life at RT and neutral pH of the small molecule thioester *S*-methyl thioacetate has been reported to be 155 days (corresponding to an expected 22% degradation after 56 days).^49^ Supported by the above observations, the hydrolysis half-life of DOT-derived thioesters was presumed to be even higher due to their hydrophobic surrounding preventing the access of water.

**Figure 1:**
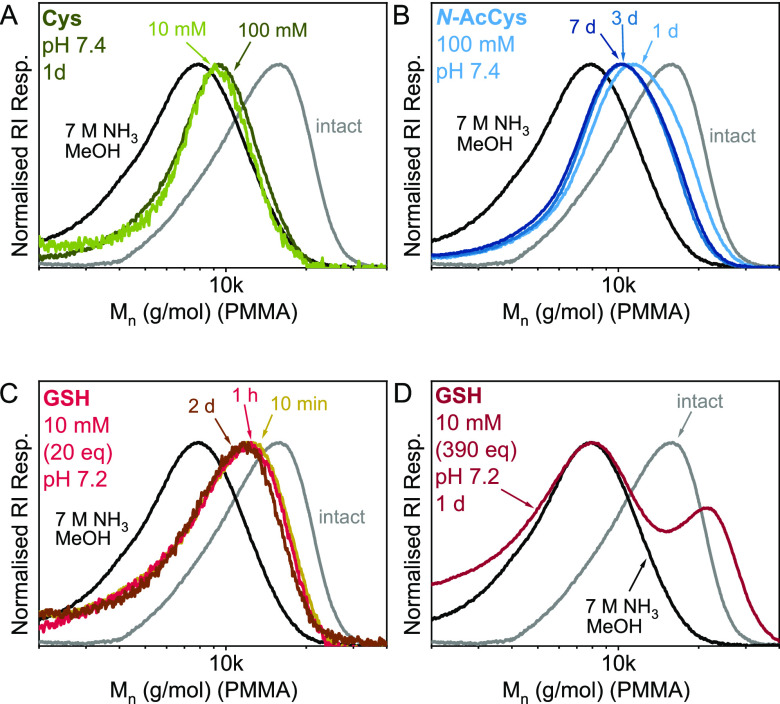
Size exclusion chromatograms of p(PEGA_218_-DOT_22_) (Table 1, entry 4) after treatment with (A) cysteine (10 mM, 100 mM) at pH 7.4 for 1 day; (B) *N*-acetylcysteine (100 mM) at pH 7.4 for 1, 3, and 7 d; (C) glutathione (10 mM, corresponding to a GSH/thioester ratio of 20:1) at pH 7.2 for 10 min, 1 h, and 2 d; and (D) glutathione (10 mM, GSH/thioester ratio of 390:1) at pH 7.2 for 1 d. For comparison, the chromatograms of the intact copolymer (gray curves) and after complete degradation through ammonolysis (black curves) are shown in each plot.

Next, p(PEGA_218_-DOT_22_) (Table 1, entry 4) was treated with cysteine at 37 °C in PBS at pH 7.4 to mimic the physiological extracellular ionic strength and pH. Gratifyingly, the degradation was judged to be complete after 1 day in both 100 mM and 10 mM cysteine solution (corresponding to thiol/thioester ratios of 200 and 20, respectively). SEC analysis of the cysteine-degraded samples (Figure 1A) showed the higher molecular weight onset and slope of the curves matching that of a control sample degraded with 7 M NH_3_ in MeOH, indicating full degradation of the thioesters. The trailing of the control sample toward lower molar masses was presumed to arise from interactions of the amide-functional polymers with the SEC column material and/or the presence of lower molar mass fragments present in the control sample (due to different workup; see Supporting Information).

On the other hand, treatment with *N*-acetylcysteine (100 mM) under the same conditions did not fully degrade p(PEGA_218_-DOT_22_). Although SEC analysis (Figure 1B) showed clear decreases in hydrodynamic size after 1 and 3 d, no additional change was observed after a total of 7 d at 37 °C, suggesting that an equilibrium fragment size had been reached after 3 d. We presumed the difference in degradation efficiency between cysteine and its *N*-acetylated cousin to be due to two reasons. First, cysteine has a relatively low p*K*_a_ (S–H) of 8.3,^50^ meaning approximately 12% of molecules are deprotonated to the reactive thiolate species at pH 7.4. The less acidic *N*-acetylcysteine, on the other hand (p*K*_a_ (S–H) = 9.5)^50^ only undergoes approximately 0.8% deprotonation at pH 7.4. Second, a difference in degradation mechanism between the two cysteine derivatives was presumed. During cysteinolysis, *S*-acylated cysteine derivatives are known to undergo an S–N shift to form the amide-functional product (Scheme 1C).^51^ While this S–N shift was shown to be reversible at low pH, the equilibrium lies firmly on the side of the amide at pH 7.4.^52^ On the other hand, the degradation with *N*-acetylcysteine lacks this additional step and the degradation (through thiol–thioester exchange, a reaction that can be slow)^49^ is therefore reversible with the expelled macromolecular thiol able to replace an *S*-acylated *N*-acetylcysteine residue and form a new polymer–SC(=O)–polymer linkage (Scheme 1D).

This reversibility during thiolysis was even more apparent when glutathione (GSH, which likewise lacks the irreversible S–N shift) was used. First, p(PEGA_218_-DOT_22_) was treated with 10 mM GSH (GSH/thioester ratio = 20:1) in phosphate buffered saline at pH 7.2 mimicking the intracellular pH and GSH concentration.^53^ The lower p*K*_a_ (S–H) (compared to *N*-acetylcysteine) of 8.6 (corresponding to 4% deprotonation at pH 7.2)^50^ makes GSH a more efficient nucleophile during polymer–SC(O)–polymer cleavage but also a better leaving group during the reverse reaction. This means that an equilibrium could be established faster than with the less acidic *N*-acetylcysteine. Indeed, a clear shift in hydrodynamic size was observed by SEC after just 10 min at 37 °C with 10 mM GSH with no additional changes observed after 1 h or 2 d (Figure 1C) The degradation was, however, judged to be incomplete based on a comparison with a sample degraded with 7 M ammonia. To push the equilibrium further toward the side of degraded polymers without increasing the pH or increasing the concentration of GSH beyond intracellular levels, a lower polymer concentration (0.128 g/L) was used with a GSH/thioester ratio of 390:1. Surprisingly, after 1 d at 37 °C, a bimodal distribution was observed in SEC analysis, see Figure 1D. The lower molar mass peak coincided with that of an ammonia-degraded control sample, although with more trailing toward lower molar masses, presumably due to the interactions of the peptide end groups of the fragments with the column material. The secondary peak belonged to a species of higher molar mass than the original polymer and did not elute at a time expected for a double molar-mass fragment formed through thiol oxidation. Degradations were done in the presence of tris(carboxyethyl)phosphine (TCEP) as a reducing agent to prevent disulfide formation (a control experiment, not shown, indicated that TCEP by itself did not cause degradation). Instead, it was presumed that the high molar mass species were formed through “reverse degradation” by polycondensation of GS–C(=O)–polymer–SH species, based on the fast thiol–thioester exchange equilibrium. Presumably, due to steric restraints^44,51^ the cleavage of thioesters in the periphery of a polymer coil is slightly faster than the cleavage of thioesters carrying long polymer chains on both sides. This makes randomly formed larger copolymers more stable toward degradation and might explain the occurrence of the bimodal distribution. While the polycondensation of degradation fragments offers potential for recycling applications and undoubtedly warrants further work beyond the current scope, we note here that employing a higher GSH/thioester ratio indeed resulted in the complete degradation of most polymer chains under conditions mimicking the intracellular pH and GSH concentration. Overall, due to the high nucleophilicity of thiolates and the irreversibility under physiological conditions, the most efficient degradation method was cysteinolysis, followed by cleavage through glutathione and *N*-acetylcysteine. Conversely, in an organic solution complete degradation could be achieved quickly by using a thiolate, for example, propane thiol in the presence of triethylamine.^35^

**Figure 2:**
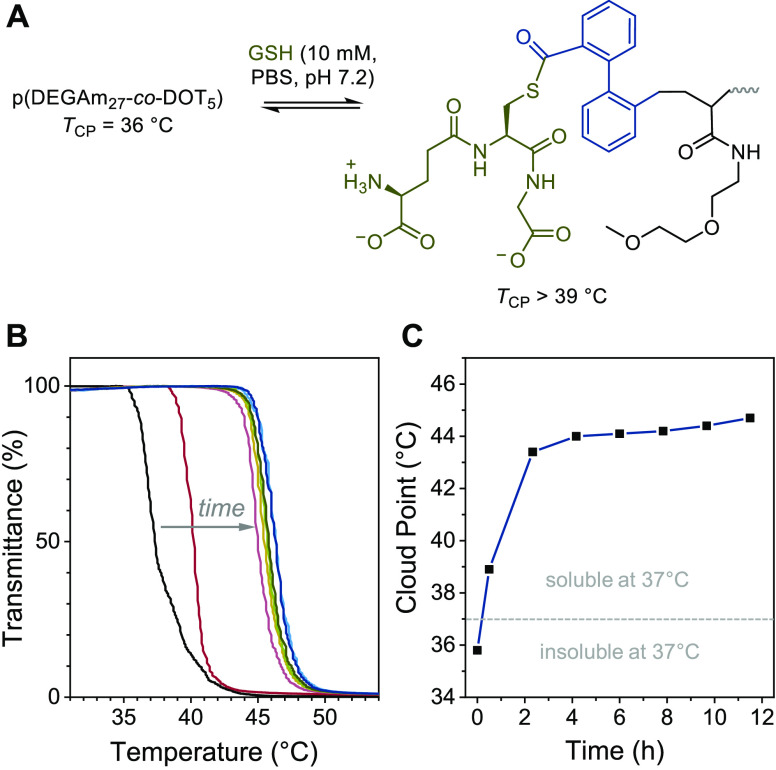
Degradation of a thermoresponsive copolymer with 10 mM glutathione: structures (A), turbidity curves after addition of glutathione (B), and change of measured cloud point versus time with a dashed line indicating core body temperature (C).

These results are significant because they represent the first example of a radically made vinyl copolymer degrading quickly (at least partially) through thiolysis at physiological pH and intracellular GSH concentration without the need for the harsh conditions required for polyester materials. The physiological concentration of free (reduced) cysteine is low (around 7.5–10 μM, i.e., 3–4 orders of magnitude lower than used here)^54^ and not expected to contribute to quick degradation of thioester backbone-functional polymers. *N*-acetylcysteine is a nontoxic dietary supplement known to increase the concentrations of thiols^55^ and therefore offers the opportunity of triggering the degradation of an injected polymer material through oral doses of this drug. GSH, on the other hand, has a high intracellular concentration (1–10 mM), roughly 3 orders of magnitude higher than its extracellular concentration.^53^ Additionally, GSH levels have been shown to be elevated in lung, breast, and gastrointestinal cancer tissues,^56^ paving the way for the selective degradation of polymer carriers in cancer tissue or following endocytosis. This degradation can be envisaged to facilitate the clearance of polymer material from the body by decreasing its size below the renal filtration threshold.^57^ Additionally, we demonstrate here that even partial equilibrium degradation through the influence of GSH evoked an insoluble–soluble transition under physiological conditions. P(DEGAm_27_-DOT_5_) (Table 1, entry 8) had a measured lower critical solution temperature (LCST) transition at 36 °C, that is, was fully water-soluble only below this temperature. The transition was fully reversible over three heating–cooling cycles (between 20–70 °C) suggesting hydrolytic stability for several hours at elevated temperatures, see Figure S2. Upon adding 10 mM GSH (pH 7.2), the measured cloud point increased within 30 min to 39 °C and reached a final value of 44 °C after 4 h, see Figure 2. Notably, this comparatively small change in transition temperature was sufficient to switch the material from insoluble to fully soluble at a core body temperature of 37 °C, promising for the release of an encapsulated payload.

### Cytotoxicity

**Figure 3:**
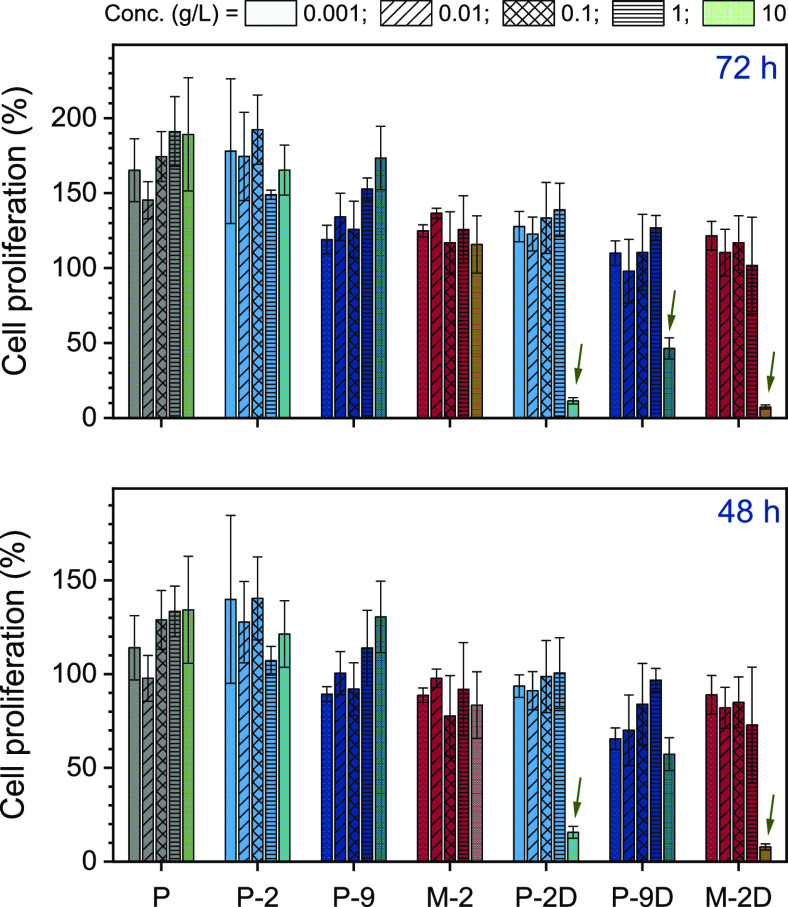
Cell proliferation (where 100% refers to the metabolic activity of a control sample not exposed to polymer solutions and 0% to no residual metabolic activity) of human pancreatic adenocarcinoma cells 48 h (bottom) and 72 h (top) after being incubated with solutions of pPEGA_247_ (P, gray), p(PEGA_0.98_-DOT_0.02_)_248_ (P-2, light blue), p(PEGA_0.91_-DOT_0.09_)_240_ (P-9, dark blue), p(DEGAm_0.98_-DOT_0.02_)_211_ (M-2, red), and after degradation with cysteine (suffix “D”) at concentrations of 0.001 g/L (dotted bars), 0.01 g/L (diagonally lined bars), 0.1 g/L (hashed bars), 1 g/L (horizontally striped bars), and 10 g/L (textured bars). Error bars show the standard deviation between three separate repetitions. Results showing toxicity are indicated by arrows.

Next, the cytotoxicity of intact polymers and their degradation fragments was assessed. The PEGA-based copolymers p(PEGA_243_-DOT_5_) and p(PEGA_218_-DOT_22_) (containing 2 and 9 mol % of thioester-functional repeat units, respectively, Table 1, entries 2 and 4), the acrylamide species p(DEGAm_206_-DOT_5_) (containing 2 mol % DOT units, Table 1 entry 5), and p(PEGA_247_) homopolymer (as a nondegradable comparison, Table 1, entry 1) were used intact and after degradation with cysteine (chosen to ensure complete degradation, which was confirmed by SEC analysis, see Figure S3). Human pancreatic adenocarcinoma cells were incubated with polymer solutions with concentrations ranging from 0.001 to 10 g/L and their metabolic activity was assessed through an Alamar Blue assay after 48 and 72 h, see Figure 3. Cell proliferations of some samples were above 100% after 72 h indicating better metabolic activity than a control not exposed to polymer solutions. Gratifyingly, the intact polymers were not cytotoxic with excellent cell proliferation even at polymer concentrations of 10 g/L. Samples degraded with cysteine showed toxicity only at concentrations of 10 g/L after 48 h (two samples) and 72 h (all three samples) (see arrows in Figure 3), with no toxicity observed at lower concentrations. In biomedical applications, polymer concentrations are typically well below 1 g/L (and the cytotoxicity of higher concentrations is commonly not assessed). It was presumed that the toxicity observed at higher concentrations was associated with the thiol end groups produced through thioester cleavage. Indeed, the literature reports that for RAFT-made pPEGA homopolymers the thiol end groups formed through aminolysis or hydrolysis of the thiocarbonylthio RAFT end groups contributed strongly to the toxicity toward mouse embryonic fibroblast cells at a concentration of 10 g/L.^58^

### Application for Drug Release

**Figure 4:**
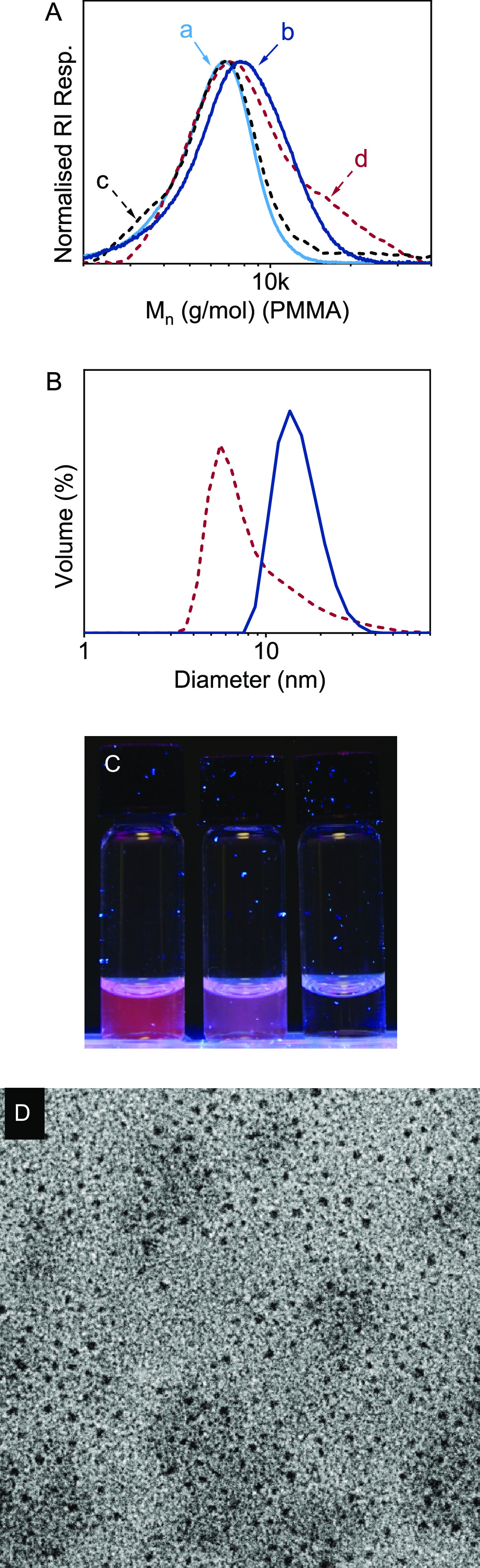
Core-degradable diblock copolymer micelles. (A) SEC elution curves of pPEGA_24_ macro-RAFT agent (*M*_n_ = 5.6 kg/mol, *Đ* = 1.13, curve a), diblock copolymer p[PEGA_24_-*block*-(DEGAm_55_-*co*-DOT_12_)] (*M*_n_ = 6.8 kg/mol, *Đ* = 1.20, curve b), and after full degradation with cysteine (*M*_n_ = 5.8 kg/mol, *Đ* = 1.14, curve c) and partial degradation with glutathione (*M*_n_ = 7.5 kg/mol, *Đ* = 1.27, curve d). (B) Volume-average size distribution measured by dynamic light scattering of intact p[PEGA_24_-*block*-(DEGAm_55_-*co*-DOT_12_)] micelles in PBS buffer (blue solid line) and after partial degradation with glutathione (dotted red line). (C) Photograph taken under UV irradiation (λ_max_ = 365 nm) of vials containing p[PEGA_24_-*block*-(DEGAm_55_-co-DOT_12_)] and Nile Red in buffer (pH = 7.2) (left), the same mixture after addition of glutathione (middle), and a control of Nile Red in PBS buffer in the absence of polymer (right). (D) Transmission electron microscopic image (width 200 nm × 200 nm) of dried intact p[PEGA_24_-*block*-(DEGAm_55_-*co*-DOT_12_)] micelles.

One of the major advantages of reversible deactivation radical polymerization over polycondensation methods is the ability to prepare diblock copolymers that self-assemble into well-defined core–shell nanoparticles. To demonstrate the potential of thioester-backbone functional polymers for the intracellular release of encapsulated drugs, a diblock copolymer p[PEGA_24_-*block*-(DEGAm_55_-*co*-DOT_12_)] was prepared through chain extension of a pPEGA_24_ macro-RAFT agent. Treatment of the copolymer with cysteine resulted in an SEC trace overlapping that of the PEGA macro-RAFT agent confirming the degradability of the DEGAm-DOT based block and indicating that the cleavage had taken place close to the block junction. Degradation with GSH, on the other hand, resulted in incomplete degradation with SEC analysis showing a broad trace encompassing species with a hydrodynamic size matching that of the macro-RAFT agent, as well as species even larger than the diblock copolymer which were presumably formed through the “reverse degradation” polycondensation process described above, see Figure 4A.

The copolymer dispersed readily in water and PBS buffer with dynamic light scattering showing a monomodal size distribution averaging 14.9 nm volume-average (10.9 nm number-average) hydrodynamic diameters at all temperatures from 20–60 °C, see Figure 4B and Figure S4. This size distribution suggested the formation of micelles with a water-insoluble (and temperature insensitive) DEGAm-DOT core, stabilized by a soluble pPEGA shell. Indeed, addition of a small quantity of Nile Red (a solvochromic dye that is insoluble in water and only fluoresces in a hydrophobic environment, including when encapsulated)^59^ gave a strong red fluorescence under UV irradiation, suggesting it had been taken up into the hydrophobic cores of micelles, see Figure 4C. Additionally, TEM analysis showed spherical objects with a measured diameter of the dried material of 4.0 ± 0.8 nm, suggesting that the larger DLS-measured diameter was the result of swelling and hydration, see Figure 4D. These observations indicated the successful formation of degradable PEG-based nanoparticles able to encapsulate a hydrophobic payload. When glutathione (10 mM) was added to a sample of diblock copolymer micelles loaded with Nile Red, the fluorescence decreased visibly within minutes, see Figure 4C, suggesting that part of the cargo had been released through disintegration of the micelles. Control experiments showed that the addition of GSH to Nile Red in water (Figure S5) or in water–THF (Figure S6) did not cause a decrease in Nile Red fluorescence. Addition of potassium persulfate solution (reported to rapidly cleave thioesters)^35^ to another sample of Nile Red-loaded micelles resulted in the immediate disappearance of fluorescence, suggesting complete release caused by the fast and selective oxidative cleavage of the thioesters, see Figure S7. When DLS analysis was performed at a sufficiently high GSH/thioester ratio (polymer concentration 0.25 g/L, 30 mM GSH, GSH/thioester ratio = 240:1), a bimodal size distribution by volume was observed including a peak at 5.6 nm volume-average diameter. This data was interpreted to show a combination of residual micelles and solvated fragments of disintegrated micelles including GSH-bound DEGAm species and the PEGA-based macroinitiator. Surprisingly, when diblock copolymer micelles at a higher concentration of 1 g/L were treated with GSH, DLS analysis showed a monomodal distribution with a volume-average diameter of 173 nm (not shown). To investigate the nature of these unexpectedly large species (which appeared to eclipse the visibility of fully soluble fragments including the solubilizing pPEGA corona), a sample of the diblock copolymer was degraded with ammonia in methanol (which allowed complete removal of excess degradant through evaporation). Similar to the GSH degraded samples, an aqueous dispersion of the residual material gave a DLS-measured volume-average hydrodynamic diameter of 142 nm (not shown). This dispersion was extracted with diethyl ether. NMR analysis of the dried organic extracts showed 2-phenylbenzamide species connected to zero or one DEGAm repeat units formed through the degradation of DOT–DOT and DOT–DEGAm–DOT sequences. On the other hand, the washed aqueous phase was found to contain soluble species accounting for >95 wt % of the original diblock copolymer material. These observations indicated that a glutathione-triggered release of an encapsulated payload from thioester core-functional micelles was indeed possible but that the specific diblock copolymer composition investigated here led to the formation of species with limited water solubility that aggregated at an initial diblock copolymer concentration of 1 g/L. It is possible that such aggregates also contributed to the toxicity observed of degraded species at high polymer concentrations.

## Conclusions

While polymers with backbone thioesters in every repeat unit are accessible through nonradical methods, the radical comonomer approach presented here allowed inserting degradable thioester units into well-known and novel PEG-based vinyl monomers and taking advantage of the low toxicity, water-solubility, and/or temperature-responsive (“smart”) behavior of these vinyl comonomer base materials. The resulting backbone thioesters were stable in aqueous solution over weeks but were rapidly cleaved in the presence of thiols. A high ratio of reactive thiolates to thioesters, governed by the thiol concentration, solution pH, and thiol p*K*_a_ value, was paramount to push the reversible thiol–thioester equilibrium toward the side of degraded species. Thus, under mimicked intracellular conditions (PBS, 10 mM glutathione, pH 7.2), efficient degradation required polymer concentrations lower than 1 g/L. “Reverse degradation” through the polycondensation of RS–C(=O)–polymer–SH species led to the formation of minor amounts of copolymers larger than the original intact polymers. Despite these limitations, glutathione-triggered degradation was shown to switch a copolymer from insoluble to soluble at body temperature and to facilitate the (partial) release of Nile Red (as a model for a hydrophobic drug) encapsulated into amphiphilic diblock copolymer micelles. Thioester-functional copolymers and their cysteinolytic degradation products were found to be nontoxic toward human pancreatic adenocarcinoma cells at concentrations up to 1 g/L. The selective thiolysis under biologically relevant conditions makes copolymers derived through thiocarbonyl addition–ring-opening (TARO) radical polymerization promising materials in the development of next-generation biomaterials for drug release and delivery.

## Supporting Information Available

Instrumentation, materials, synthesis of di(ethylene glycol) methyl ether acrylamide (DEGAm), additional SEC and turbidity data, photographs showing the release of Nile Red, and of control experiments (PDF)
