# Supplementary material for: Biocompatibility and Physiological Thiolytic Degradability of Radically Made Thioester-Functional Copolymers: Opportunities for Drug Release
Source: Biomacromolecules. 2022 Apr 26;23(5):2031–9. doi: 10.1021/acs.biomac.2c00039 (PMC9092349; doi:10.1021/acs.biomac.2c00039)
Supplement: Supplementary file 1 — bm2c00039_si_001.pdf [file bm2c00039_si_001.pdf]

—Supporting Information—

Biocompatibility and Physiological Thiolytic Degradability of Radically-made Thioester-functional Copolymers: Opportunities for Drug Release

*Nathaniel M. Bingham,[a,+] Qamar un Nisa,[a,+] Priyanka Gupta,[b] Neil P. Young,[c] Eirini Velliou,[b,d] and Peter J. Roth[a,]**

[a] Department of Chemistry, School of Chemistry and Chemical Engineering, University of Surrey, Guildford, Surrey, GU2 7XH, UK

[b] Department of Chemical and Process Engineering, School of Chemistry and Chemical Engineering, University of Surrey, Guildford, Surrey, GU2 7XH, UK

[c] Holder Building, Department of Materials, University of Oxford, Parks Road, Oxford, OX1 3PH, UK

[d] Centre for 3D Models of Health and Disease, UCL-Division of Surgery and Interventional Science, Charles Bell House, 43–45 Foley Street, Fitzrovia, London, W1W 7TY, UK

* corresponding author email address p.roth@surrey.ac.uk

[+] equal contribution of both authors

Contents

Experimental Section 2

Results and Discussion 3

References 5

Experimental Section

Instrumentation

NMR spectroscopic measurements were performed on 400 or 500 MHz Bruker instruments in 5 mm NMR tubes. Residual solvent signals of $CHCl_3$ (δ_H = 7.26 ppm, δ_C = 77.2 ppm) were used as references.

Fourier transform infrared spectroscopy (FT-IR) was performed on a Perkin Elmer FT-IR spectrometer under attenuated total reflection (ATR).

Size exclusion chromatography (SEC) was performed on a Viscotek GPCMax VE 2001 setup with three linear 7.5×300 mm PLgel mixed-D columns connected to a Viscotek VE3580 refractive index (RI) detector and a Malvern 270 dual detector (viscometer and light scattering). The instrument operated at 35 °C with tetrahydrofuran (THF) containing 250 ppm BHT as mobile phase at a flow rate of 1.0 mL×min^{-1}. The system was calibrated using a series of narrow molecular weight distribution PMMA standards with molecular weights ranging from 5 kg×mol^{-1} to 298 kg×mol^{-1}.

LCST cloud points were determined by temperature-dependant turbidity measurements on a Thermo Scientific Evolution 201 UV-Visible Spectrometer equipped with a Peltier control and cooling unit (PCCU1) in plastic cuvettes of 10 mm path length at a wavelength of 550 nm with heating/cooling rates of 1 °C/min. Polymer concentrations were 5 g/L. For clear solutions the baseline was corrected to zero absorbance, A. Transmittance, $T = 10^{-A}$, was plotted against temperature, and cloud points were determined at T = 95%.

TEM images were recorded on a JEOL JEM-2100 operating at a maximum accelerating voltage of 200 kV. A drop of micellar solution (conc = 1 g/L) was placed on a carbon-coated copper grid (300 mesh Cu grid, Agar Scientific). Excess solution was wicked away from the grid with filter paper. No staining was applied. Samples were left to dry at RT.

Materials

All reagents were purchased from Sigma-Aldrich and used as received, unless noted otherwise. 2,2'-Azobis(isobutyronitrile) (AIBN) was recrystallized from methanol and stored in a freezer. *N*-isopropylacrylamide (NIPAm) was recrystallized from toluene–petroleum ether (50:50) and stored in a freezer. Liquid monomers were deinhibited by passing through a column of basic alumina immediately before use. The synthesis of dibenzo[c,e]oxepin-5(7*H*)-thione (DOT)[1] was previously described. Di(ethylene glycol) methyl ether amine was prepared as previously described[2] and stored in a fridge for 11 years except during rail transport from Sydney to Perth and air transport from Perth to Guildford.

Di(ethylene glycol) methyl ether acrylamide (DEGAm)

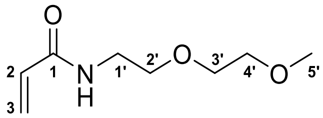

DEGAm

$C_8H_{15}NO_3$

173.21 gmol^{-1}

Di(ethylene glycol) methyl ether amine (2.50 g, 21.0 mmol, 1.1 eq.) was dissolved in DCM (30 mL) and the mixture cooled to 0 °C before a dropwise addition of triethylamine (2.95 mL, 21.0 mmol, 1.1 eq) dissolved in DCM (10 mL). To the reaction mixture acryloyl chloride (1.55 mL, 19.1 mmol, 1.0 eq) dissolved in DCM (10 mL) was added dropwise. Upon completion, the mixture was allowed to warm to RT. It was left to react for a further 2 hours and then was quenched with water (20 mL) and acidified to pH 2.0 with HCl (2 M). The organics were removed, and the aqueous phase was extracted with DCM (2 × 20 mL), combined, dried ($MgSO_4$) and concentrated *in vacuo* to afford a yellow oil (2.40 g, 73%).

^{1}H NMR (400 MHz, $CDCl_3$) δ 6.27 (br s, 1H, **NH**), 6.25 (dd, *J* = 17.0, 1.6 Hz, 1H, **2**), 6.08 (dd, *J* = 17.0. 10.2 Hz, 1H, **3**), 5.60 (dd, *J* = 10.2, 1.5 Hz, 1H, **3**), 3.62 – 3.51 (m, 8H, **1'-4'**), 3.37 (s, 3H, **5'**).

^{13}C NMR (101 MHz, $CDCl_3$) δ 165.64 (**1**), 131.00 (**2**), 126.37 (**3**), 71.92 (**2'/3'/4'**), 70.28 (**2'/3'/4'**), 69.87 (**2'/3'/4'**), 59.10 (**5'**), 39.32 (**1'**).

Results and Discussion

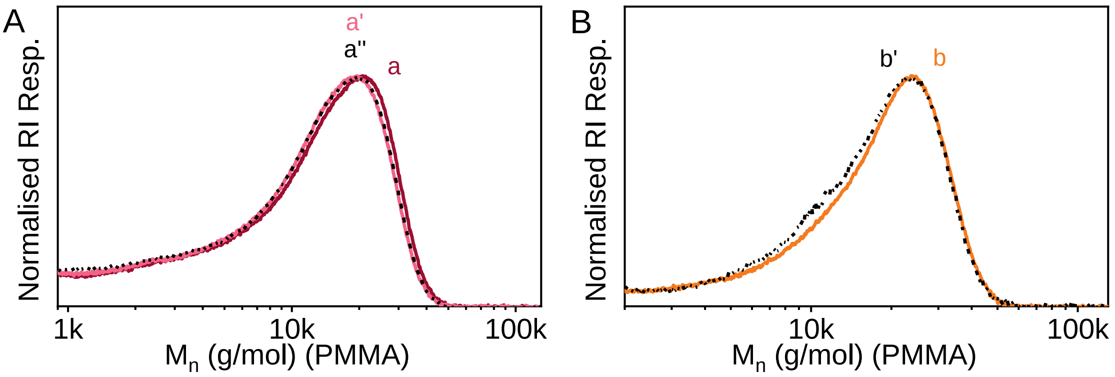

Figure S1. Stability of thioester backbone-functional copolymers in aqueous solution: SEC traces of A) p(PEGA$_{243}$-DOT$_5$) intact (curve a), after being dissolved (conc. = 3 g/L) in water (curve a') and phosphate buffered saline (pH = 7.4) (curve a'') at RT for 7 days and B) p(PEGA$_{235}$-DOT$_{11}$) intact (curve b) and after being dissolved in water (conc. = 3 g/L) for 56 days at RT.

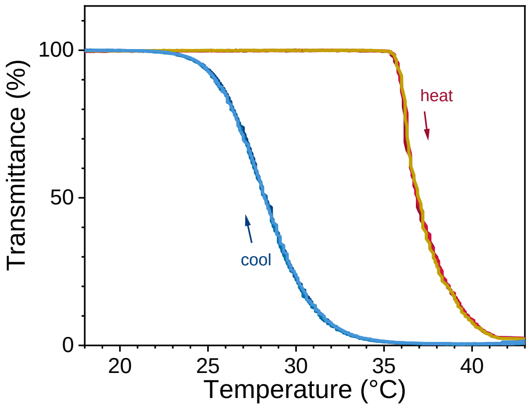

Figure S2. Three successive heating–cooling cycles measured of p(DEGAm$_{27}$-DOT$_5$) in phosphate buffered saline (5 g/L) showing reversibility of the LCST transition in the absence of GSH.

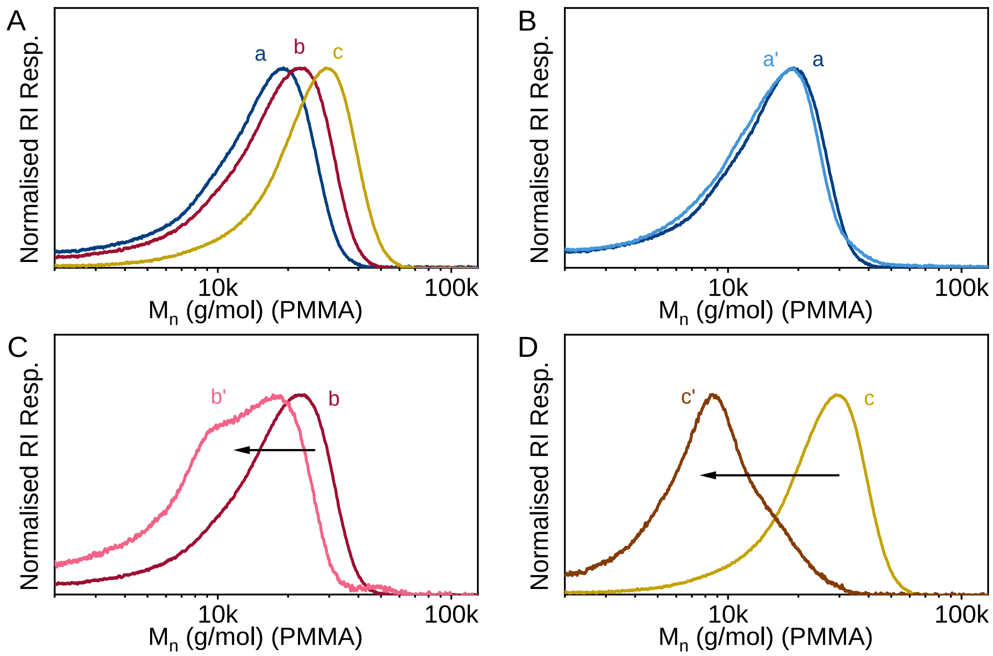

Figure S3. SEC elugrams of intact pPEGA$_{247}$ (labelled a), p(PEGA$_{243}$-DOT$_5$) (labelled b), and p(PEGA$_{218}$-DOT$_{22}$) (labelled c) (graph A) and after treatment with cysteine (B–D, primed labels). Although all copolymers had comparable degrees of polymerization, their hydrodynamic size in THF increased with an increasing DOT content (plot A). The PEGA homopolymer was virtually unchanged through the cysteine treatment (plot B). The shift between intact and degraded is species is largest for the highest DOT content (plots B–D). The associated measured molar masses and dispersities are given in the below table.

	**pPEGA$_{247}$**	**p(PEGA$_{243}$-DOT$_5$)**	**p(PEGA$_{218}$-DOT$_{22}$)**	**p(DEGAm$_{206}$-DOT$_5$)**
**Intact**	(a) M_n = 11.5 kg/mol<br>$Đ$ = 1.36	(b) M_n = 14.1 kg/mol<br>$Đ$ = 1.32	(c) M_n = 20.7 kg/mol<br>$Đ$ = 1.24	not visible
**After cysteine treatment**	(a’) M_n = 11.3 kg/mol<br>$Đ$ = 1.35 (control)	(b’) M_n = 9.1 kg/mol<br>$Đ$ = 1.43	(c’) M_n = 7.0 kg/mol<br>$Đ$ = 1.34	not visible

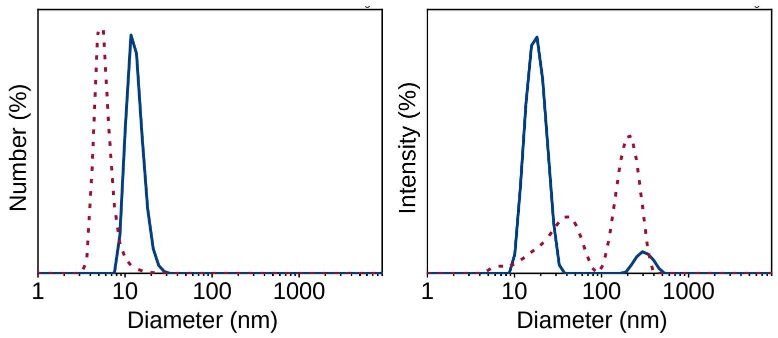

Figure S4. Number (left) and intensity (right) average sizes measured by dynamic light scattering of intact p[PEGA$_{24}$-*block*-(DEGAm$_{55}$-*co*-DOT$_{12}$)] micelles in PBS buffer (blue solid lines) and after degradation with glutathione (dotted red lines). Samples were not filtered; the intensity-average data overemphasises large aggregates.

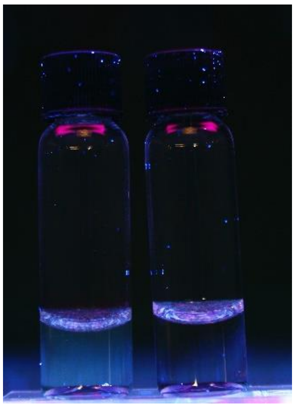

Figure S5. Photograph taken under UV irradiation (λ_{max} = 365 nm) of vials containing Nile Red and GSH in buffer (pH 7.2) (left) and Nile red only in buffer (pH 7.2) (right, same sample as shown in photograph in Figure 4C). The GSH-containing sample shows some very faint fluorescence indicating that the fluorescence *decrease* observed during the release experiment was not caused through GSH–Nile red interactions.

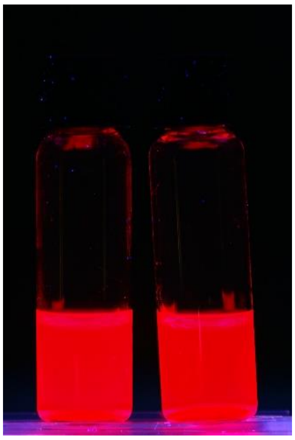

Figure S6. Photograph taken under UV irradiation (λ_{max} = 365 nm) of vials containing Nile red and GSH in water (pH 7.2)–THF (50:50 by volume) (left)and Nile red only in the same solvent mixture without GSH (right). Note that Nile red is virtually insoluble in water; the addition of THF allows the observation of fluorescence, unlike the samples in Figure S5.The comparison confirms that GSH does not influence or decrease the fluorescence of Nile red.

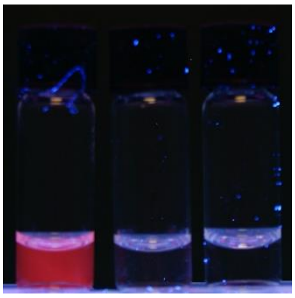

Figure S7. Photograph taken under UV irradiation (λ_{max} = 365 nm) of vials containing p[PEGA$_{24}$-*block*-(DEGAm$_{55}$-*co*-DOT$_{12}$)] and Nile red in buffer (pH = 7.2) (left), the same mixture after addition of 10 mM potassium persulfate (middle), and a control of Nile Red in PBS buffer in the absence of polymer (right). A separate experiment (not shown) showed that while the addition of persulfate to Nile Red in water–THF (1:1) changed the emission wavelength of Nile Red, it did not stop the fluorescence.

References

1. Bingham, N. M.; Roth, P. J., Degradable vinyl copolymers through thiocarbonyl addition-ring-opening (TARO) polymerization. *Chem. Commun. (Camb)* **2019,** *55* (1), 55-58.
2. Chua, G. B. H.; Roth, P. J.; Duong, H. T. T.; Davis, T. P.; Lowe, A. B., Synthesis and Thermoresponsive Solution Properties of Poly[oligo(ethylene glycol) (meth)acrylamide]s: Biocompatible PEG Analogues. *Macromolecules* **2012,** *45* (3), 1362-1374.
